# The Application of EEG Mu Rhythm Measures to Neurophysiological Research in Stuttering

**DOI:** 10.3389/fnhum.2019.00458

**Published:** 2020-01-10

**Authors:** David Jenson, Andrew L. Bowers, Daniel Hudock, Tim Saltuklaroglu

**Affiliations:** ^1^Department of Speech and Hearing Sciences, Elson S. Floyd College of Medicine, Washington State University, Spokane, WA, United States; ^2^Epley Center for Health Professions, Communication Sciences and Disorders, University of Arkansas, Fayetteville, AR, United States; ^3^Department of Communication Sciences and Disorders, Idaho State University, Pocatello, ID, United States; ^4^College of Health Professions, Department of Audiology and Speech-Pathology, University of Tennessee Health Science Center, Knoxville, TN, United States

**Keywords:** stuttering, mu rhythm, sensorimotor integration, speech production, speech perception, working memory, internal models, basal ganglia

## Abstract

Deficits in basal ganglia-based inhibitory and timing circuits along with sensorimotor internal modeling mechanisms are thought to underlie stuttering. However, much remains to be learned regarding the precise manner how these deficits contribute to disrupting both speech and cognitive functions in those who stutter. Herein, we examine the suitability of electroencephalographic (EEG) mu rhythms for addressing these deficits. We review some previous findings of mu rhythm activity differentiating stuttering from non-stuttering individuals and present some new preliminary findings capturing stuttering-related deficits in working memory. Mu rhythms are characterized by spectral peaks in alpha (8–13 Hz) and beta (14–25 Hz) frequency bands (mu-alpha and mu-beta). They emanate from premotor/motor regions and are influenced by basal ganglia and sensorimotor function. More specifically, alpha peaks (mu-alpha) are sensitive to basal ganglia-based inhibitory signals and sensory-to-motor feedback. Beta peaks (mu-beta) are sensitive to changes in timing and capture motor-to-sensory (i.e., forward model) projections. Observing simultaneous changes in mu-alpha and mu-beta across the time-course of specific events provides a rich window for observing neurophysiological deficits associated with stuttering in both speech and cognitive tasks and can provide a better understanding of the functional relationship between these stuttering symptoms. We review how independent component analysis (ICA) can extract mu rhythms from raw EEG signals in speech production tasks, such that changes in alpha and beta power are mapped to myogenic activity from articulators. We review findings from speech production and auditory discrimination tasks demonstrating that mu-alpha and mu-beta are highly sensitive to capturing sensorimotor and basal ganglia deficits associated with stuttering with high temporal precision. Novel findings from a non-word repetition (working memory) task are also included. They show reduced mu-alpha suppression in a stuttering group compared to a typically fluent group. Finally, we review current limitations and directions for future research.

## Introduction

Decades of research have converged on two distinct yet related neural mechanisms implicated in the neurophysiology of stuttering. These mechanisms are: (1) the basal ganglia mechanism that helps provide timing cues for speech and inhibit irrelevant neural information (Alm, 2004; Civier et al., 2010; Chang and Zhu, 2013; Chang et al., 2019); and (2) the sensorimotor system that helps guide articulatory movements *via* internal modeling (Max et al., 2004; Loucks and De Nil, 2006; Mersov et al., 2016; Chang et al., 2019). Despite the identification of compromise in these mechanisms, much remains to be understood regarding the neurophysiological breakdowns in these mechanisms that result in overt stuttering behaviors, how these breakdowns can be overcome to reinstate fluency and, how they may contribute to differences in cognitive function that are associated with stuttering. The developmental, intermittent and highly variable nature of stuttering combined with a limited temporal resolution that is inherent to some functional neuroimaging techniques have created challenges in separating trait- from state-related patterns of neural activity (Belyk et al., 2015, 2017) and thus, the separation of cause and effect when interpreting data.

To overcome the development barrier, neuroimaging data must continue to be acquired from children as close to the onset of stuttering as possible (Chow and Chang, 2017). Other barriers may be overcome by careful experimental design and the use of high temporal resolution neuroimaging tools such as electroencephalography (EEG) and magnetoencephalography (MEG). To identify mechanisms underlying stuttering in speech production, it is necessary to eliminate, or at least control for, the effects of overt stuttering on neural activation. By the same logic, it also is necessary to preclude the use of fluency enhancing techniques (e.g., speech restructuring strategies, choral speech, delayed auditory feedback, etc). As such, the best means of identifying trait-related differences in speech is to compare neural activity from spontaneously fluent utterances in people who stutter (PWS) and matched typically fluent speakers (TFSs; Jenson et al., 2018; Mersov et al., 2018). However, it should be noted even the perceptually fluent speech of PWS might be influenced by effects of the underlying pathology and therefore, interpretations need to be made cautiously (Armson and Kalinowski, 1994; Belyk et al., 2015).

Enhanced understanding of stuttering neurophysiology may be acquired through the study of related, non-speech cognitive functions. Perceiving speech has long been known to activate the same sensorimotor mechanisms that are involved in speech production (Callan et al., 2010; Bowers et al., 2013), with activation levels that typically correlate with task difficulty (Szenkovits et al., 2012). Thus, increased sensorimotor activity appears to be associated with cognitive resource allocation (e.g., attention and working memory) that increases to support more difficult tasks (e.g., discriminating in noisy backgrounds). The activity is likely because the same dorsal stream sensorimotor regions involved in speech production also can subserve general cognitive mechanisms such as attention and phonological working memory (PWM). Internal modeling mechanisms that drive sensorimotor integration are also strongly implicated in attention (Schröger et al., 2015) and working memory (Hickok et al., 2003; Buchsbaum and D’Esposito, 2019). Given the functional overlap, it perhaps does not seem surprising that the effects of stuttering can transcend speech production and impact cognitive function. Thus, the growing behavioral evidence of these cognitive effects in PWS (Byrd et al., 2015; Eggers and Jansson-Verkasalo, 2017; Eichorn et al., 2018; Coalson et al., 2019), make it necessary to understand their neural correlates. An added advantage of studying the effects of stuttering on cognitive function is that it can provide a valuable window into understanding how sensorimotor function differs in PWS without the potentially contaminating effects of overt stuttering.

### Improving Temporal Resolution

One reason for continued limitations in understanding the neurophysiology of stuttering is the dearth of temporally precise neuroimaging data. Sensorimotor activity for speech begins prior to the onset of production as the speech mechanism prepares for movement. It is maintained throughout production and even persists after speech movements are complete as the system resets. All these different phases of motor execution contain potentially valuable information about the nature of sensorimotor compromise associated with stuttering. However, without precise temporal resolution and the ability to map neural activity to articulator movement, it is not possible to discern changes in sensorimotor control as they occur over the time course of speech production. Similarly, in perceptual tasks, improved temporal precision can differentiate between the contributions of various cognitive processes such as attention and working memory. EEG offers the temporal resolution necessary to address the dynamics of sensorimotor integration described above. Applied to stuttering, a number of recent studies have examined event-related potentials (ERPs) in speech motor preparation (Daliri and Max, 2015, 2018; Vanhoutte et al., 2016; Ning et al., 2017). Other studies have examined oscillatory power within specific frequency bands. For example, measures of alpha rhythm (8–13 Hz) power have been used to compare emotional reactivity in children who stutter (Arnold et al., 2011). Beta rhythms (15–25 Hz) are also receiving considerable attention as they are thought to encode information about motor-to-sensory predictions (e.g., forward models) and are particularly sensitive to temporal variability in the auditory domain (Fujioka et al., 2015). Beta power differences related to stuttering have been observed in a number of studies (Joos et al., 2014; Etchell et al., 2016; Mersov et al., 2016; Mock et al., 2016; Sengupta et al., 2017). Given the presence of stuttering-related differences across alpha and beta bands, our labs have conducted a series of studies (“Speech Production” “Auditory Discrimination Tasks” and “Phonological Working Memory” sections below) focused on the EEG mu rhythm which is characterized by the power in both alpha and beta frequencies.

### EEG Mu Rhythm

Mu rhythms have been observed in raw EEG traces since at least the 1950s (Gastaut and Bert, 1954). They are typically characterized by a Rolandic (sensorimotor) source that is proximal to sites of integration for two basal ganglia loops involved in motor control (Dillon and Pizzagalli, 2007). Thus, power fluctuations in the mu rhythm may be influenced by both basal ganglia and sensorimotor functioning. Further, power in mu rhythms is highly sensitive to both movement-related and cognitive tasks. Traditional EEG measures often continue to define the mu rhythm within alpha frequencies (Pineda, 2005; Fox et al., 2016). However, since 1989 (Tiihonen et al., 1989; Taniguchi et al., 2000), MEG studies have been able to identify mu rhythms with single dipolar sources that include a smaller amplitude beta (15–25 Hz) peak, in addition to the traditionally observed and dominant alpha peak (Jones et al., 2009; Cheyne, 2013). Some researchers have claimed that the beta peak is a functionally non-distinct simple harmonic of the alpha band, based on observations that activity in the two bands is often highly correlated, especially in movement studies (Carlqvist et al., 2005; Brismar, 2007). Others acknowledge the importance of beta activity when looking at movement, but do not consider beta frequencies as part of the mu rhythm (McFarland et al., 2000; Hobson and Bishop, 2016).

However, there now exists ample evidence to support notions of unified mu rhythms consisting of both alpha and beta peaks with distinct yet functionally related responsivity patterns. Though mu rhythms can often be mapped to single dipole sources within sensorimotor cortex, when filtered into constituent frequencies, alpha bands tend to map to post-central sources, whereas beta bands map to precentral sources (Hari et al., 1997; Jurkiewicz et al., 2006; Ritter et al., 2009). However, perhaps most importantly, power in alpha and beta bands of the mu rhythm (henceforth mu-alpha and mu-beta) does not change at the same rate in movement studies (Hari et al., 1997; Hari, 2006; Stolk et al., 2019) and power in the two frequency bands can completely dissociate in cognitive studies (e.g., speech perception), clearly suggesting a functional distinction (Bowers et al., 2013; Brinkman et al., 2014; Jenson et al., 2014).

### EEG Mu Rhythms Identified *via* Independent Component Analysis

Rather than using traditional channel-based measures of mu-alpha power, our labs have conducted a series of studies using independent component analysis (ICA) to identify mu rhythms (i.e., mu components) from raw EEG data. ICA is a blind source separation technique which assumes the underlying source signals are statistically independent and mix linearly at the level of the scalp (Stone, 2002). The application of ICA to scalp-recorded signals helps to overcome some of the weaknesses of EEG as a brain-imaging tool (Onton et al., 2006; Delorme et al., 2012). First, sources of neural activity identified by ICA are temporally independent and spatially fixed. Therefore, they are not influenced by volume conduction which is inherent to channel-based EEG measures. Second, ICA acts as an excellent filter for separating neural activity from muscle artifact. This attribute can be particularly valuable as myogenic components (e.g., from speech articulators) can be identified, such that neural activity can be mapped to muscle movement in speech production tasks (Jenson et al., 2014, 2018). Third, the use of realistic three-dimensional head models allows neural components identified through ICA to be back-projected to hypothesized cortical sources. Though spatial resolution may never reach the level of functional magnetic resonance imaging (fMRI), the use of more dense electrode arrays and individual head models provide source localizations with accuracy on the level of 15 mm^3^ (Mégevand et al., 2014; Sohrabpour et al., 2015), that combined with the spectral information and excellent temporal resolution, provide an effective means of mapping neural activity to behavior.

### Spectral and Time-Frequency Analyses

EEG mu components identified *via* ICA are characterized by spectral peaks in both alpha and beta bands (Bowers et al., 2013; Jenson et al., 2014; Denis et al., 2017). This spectral characteristic is the primary heuristic for identification of mu rhythms, with localization to canonical sensorimotor regions serving as a confirmation of mu component identification following ICA. However, in the absence of depth recordings for comparison, it is impossible to categorically exclude the influence of non-sensorimotor sources of noise. Nonetheless, given the relative ubiquity within the field of cognitive neuroscience of using ICA to identify neural sources from scalp-recorded EEG data (over 1,500 studies listed in Google Scholar) we are confident that this represents a valid means for capturing sensorimotor activity. Once identified, basic spectral information (e.g., peak frequency and amplitude) can be compared across experimental conditions or between experimental groups. To this end, EEG spectral information has proven to be useful in identifying conditions such as dyslexia (Papagiannopoulou and Lagopoulos, 2016) and Parkinson’s disease (Caviness et al., 2016).

EEG mu rhythm spectra reflect the average power across frequencies during the time interval measured (i.e., an event). However, the clear advantage of EEG when measuring neural activity is the ability to perform time-frequency decomposition analyses. Time-frequency decomposition references spectral power across the time course of an event to the spectral power recorded during a (silent) baseline period to reveal fluctuations in oscillatory power known as event-related synchronization (ERS) and event-related desynchronization (ERD). Synchronization (higher oscillatory power) is typically interpreted as cortical inhibition whereas desynchronization (lower oscillatory power) is interpreted as cortical excitation (i.e., release from inhibition; Pfurtscheller and Lopes da Silva, 1999; Neuper and Pfurtscheller, 2001; Neuper et al., 2006). The ability to map neural activity in time is particularly useful in cognitive studies where it is important to identify attentional mechanisms that precede an event and working memory contributions that follow an event. Similarly, in motor tasks (such as speech production) neural activity can be traced from motor preparation, through the course of execution, and following execution as the system resets. In “Mu-alpha and Mu-beta in Movement and Cognitive Tasks” section below, we describe responsiveness patterns of mu rhythms in various tasks that we believe make them well-suited for stuttering research.

### Mu-alpha and Mu-beta in Movement and Cognitive Tasks

Table 1 summarizes some general findings from studies in our lab (Bowers et al., 2013, 2019; Jenson et al., 2014; Saltuklaroglu et al., 2017; Thornton et al., 2019) showing response patterns of mu-alpha and mu-beta in speech production and auditory discrimination tasks. Interpretations of the activity are also provided with further elaboration in “Mu-alpha and Mu-beta Responses in Movement” and “Mu-alpha and Mu-beta Responses in Cognitive Tasks” sections.

**Table 1:** Descriptions and tentative interpretations of typical mu-alpha and mu-beta response patterns across time in movement and cognitive tasks from experiments in our lab (Bowers et al., 2013; Jenson et al., 2014; Saltuklaroglu et al., 2017; Thornton et al., 2019).

Typical response patterns observed over time with underlying processes				
Task	Frequency band	Before	During	After
**Motor**		
	Mu-alpha	ERD Preparatory evaluation of sensory feedback	ERD Sensory feedback processing Primary somatosensory response	ERS (expected)^1^ Sensorimotor reset
	Mu-beta	ERD Preparatory forward modeling	ERD Forward modeling Primary motor response	ERS (expected) Sensorimotor reset
**Cognitive**		
	Mu-alpha	ERS Inhibitory response supporting attentional allocation	ERD Sensory to motor transformations Consistent with mirror neuron activity^2^	ERD Inverse modeling supporting working memory retention of stimuli
	Mu-beta	ERD Forward modeling supporting attention through predictive coding	ERD Stimulus processing Evaluation of prediction	ERD Forward modeling supporting working memory retention of stimuli

*^1^To date, our labs have not measured mu oscillations in this time period. However, ERS is predicted based on extant literature showing rebound following movement. ^2^Though our findings are not interpreted as evidence of mirror neuron activity, mu alpha ERD observed during stimulus presentation only might be considered evidence of a mirror neuron response*.

#### Mu-alpha and Mu-beta Responses in Movement

Many movement studies have demonstrated that mu-alpha typically localizes to post-central gyrus (Hari et al., 1997) and begins to desynchronize prior to movement, continues to desynchronize more strongly during movement, and then resynchronizes as it rebounds past baseline power immediately following movement (Hari et al., 1997; Pfurtscheller and Lopes da Silva, 1999; Hari, 2006). Fluctuations in mu-alpha power prior to and following the movement clearly indicate sensitivity to sensorimotor processing. This is corroborated by findings of mu-alpha desynchronization in the absence of movement to motor imagery tasks along with visual and auditory perception tasks (e.g., speech) that convey movement. Such findings have typically been interpreted as mu-alpha desynchronization indexing sensory-to-motor feedback. However, in real movement tasks, the strongest mu-alpha suppression, found during movement, is thought to capture a primary somatosensory response in addition to sensorimotor feedback (Jenson et al., 2018).

Mu-beta shows very similar response properties in movement tasks to mu-alpha, with slight differences in the timing of pre-movement desynchronization and post-movement rebound (Hari et al., 1997; Hari, 2006). Also, similar to mu-alpha, mu-beta desynchronizes in response to motor imagery (McFarland et al., 2000) and in visual or auditory perception tasks that represent or imply biological movement. Consistent with sources in pre-central gyrus, mu-beta desynchronization is associated with motor activity (Hari et al., 1997; Hari, 2006). In the absence of movement, it is thought to capture motor-to-to sensory transformations (i.e., forward models that are predictions of sensory consequences and compared to available feedback). Given that stuttering is hypothesized to be related to weak/unstable forward modeling (Max et al., 2004), mu-beta fluctuations in speech are likely to continue to prove sensitive to influences of stuttering (Jenson et al., 2018). However, analogous to mu-alpha, during movement, the strongest mu-beta desynchronization is thought to result from both a primary motor combined with the sensorimotor response.

**Figure 1:**
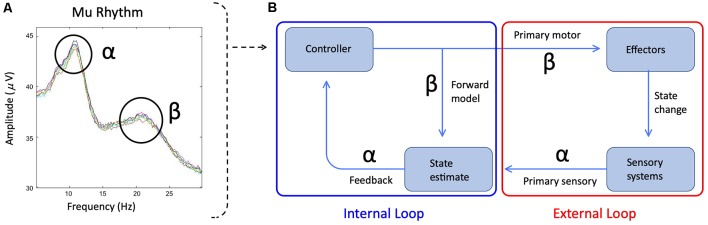
**(A)** The spectral plot of mu rhythm with alpha and beta symbols identifying the frequency peaks. Plot derived from data presented in Jenson et al. (2014). **(B)** Simplified schematic of State Feedback Control with the internal sensorimotor loop outlined in blue and the external primary motor/sensory loop outlined in red. Alpha and beta symbols indicate the sensitivity of mu bands to the distinct internal and external loop processes. Within the internal loop, mu-beta captures forward models, which represent sensory predictions of the upcoming motor plan and are encoded in projections from the premotor cortex to auditory and somatosensory cortices. Following a comparison between forward model predictions and sensory targets in auditory and somatosensory cortices, any mismatch is mapped onto corrective motor commands and returned to the premotor cortex *via* an inverse model (encoded in mu-alpha) for ongoing motor planning. Within the external loop, mu-beta encodes the primary motor response, while mu-alpha encodes sensory feedback to the premotor cortex based on the available reafference.

Based on the descriptions above, in movement tasks including speech, both mu-alpha and mu-beta desynchronization likely capture primary somatosensory and motor responses respectively during movement concomitantly with sensorimotor responses during and surrounding (i.e., preceding and following) the movement. In the context of speech production, the contributions to mu desynchronization may be akin to those from internal and external loops, with internal loops representing the sensorimotor contributions and the external looping representing the primary motor and somatosensory feedback contributions (Houde and Nagarajan, 2011, see Figure 1). Thus, when making comparisons of mu activity from motor tasks, it is necessary to control as much as possible for the movement to ensure that primary motor/somatosensory contributions to mu desynchronization are similar and therefore, differences observed can be attributed to sensorimotor function. For this reason, making comparisons of mu activity in stuttered and fluent speech may prove difficult. Even when controlling for primary motor/somatosensory contributions (e.g., within fluent speech), robust contributions to mu desynchronization from primary somatosensory and motor responses to the signal may decrease sensitivity in contrasts of sensorimotor activity (Jenson et al., 2018).

Consequently, measurements of mu activity prior to and following speech production are likely to provide the best measures of sensorimotor activity. Pre-movement beta oscillations are influenced by anticipation errors and uncertainty (Engel and Fries, 2010; Torrecillos et al., 2015; Palmer et al., 2016), whereas post-movement beta rebound (resynchronization) is thought to provide an index of error evaluation from the preceding movement (i.e., uncertainty; Tan et al., 2014), both of which may be influenced by stuttering. Further insight into stuttering also may be gleaned by using covert (imagined) speaking tasks that recruit the sensorimotor system without the need for overt production (Tian et al., 2016) or, the use of cognitive tasks which are known to recruit sensorimotor function.

#### Mu-alpha and Mu-beta Responses in Cognitive Tasks

Links between speech-related motor and cognitive processes have been investigated since the controversial Motor Theory of Speech Perception (Liberman et al., 1967). The discovery of mirror neurons linking perception to action provided some support for this theory (Rizzolatti and Arbib, 1998). However, it is now generally accepted that speech perception does not entail an obligatory motor response, though when observed, motor activity tends to increase with perceptual task demands (Szenkovits et al., 2012). In addition, temporally sensitive measures have demonstrated that motor activity in speech perception can occur prior to and following perception, suggesting that it plays a larger role than simply a direct mirror neuron-induced sensory-to-motor transformation that is observed only while speech is being perceived (Jenson et al., 2014). Thus, it is becoming clearer that motor activity observed in perception is related to sensorimotor function. The sensorimotor system alongside the basal ganglia, and in coordination with the prefrontal cortex appear to engage prior to and following perception to support cognitive processes (i.e., attention and working memory) in which perception is grounded (Heald and Nusbaum, 2014).

Heightened attention to a task is often marked by early beta desynchronization prior to stimulus processing. Similar to interpretations of mu-beta desynchronization in movement, mu-beta desynchronization in cognitive tasks is considered an indicator of top-down forward modeling used to make predictions about forthcoming stimuli (Arnal and Giraud, 2012). Interestingly beta fluctuations in cognitive tasks are influenced by auditory input, and especially to sound omissions and changes in the timing of auditory stimuli (Fujioka et al., 2015). Attentional mechanisms also influence alpha rhythms including mu-alpha oscillations. Early alpha synchronization, conveying cortical inhibition, is often observed in cognitive tasks. Inhibition is thought to reflect active inhibition of information that is irrelevant to a task or, of cortical regions that are not involved in tasks (Jensen and Mazaheri, 2010; Jenson et al., 2014). When observed in mu-alpha, it may be considered an indicator of the basal ganglia exerting inhibitory influences on sensorimotor processes (Bönstrup et al., 2015). Access to inhibitory mechanisms such as these may be particularly useful when applied to stuttering, which is thought to be associated with reduced inhibitory capacities. Given the oscillatory patterns described above, mu activity in early attentional mechanisms is particularly interesting. It is possible to observe a clear dissociation between mu-alpha (synchronization) and mu-beta (desynchronization) across time, showing how these functionally distinct bands of the same rhythm contribute to attentional mechanisms *via* cooperative inhibition and prediction mechanisms. As forward modeling, inhibitory processes, and attentional mechanisms are implicated in stuttering, it is highly likely that measurements from this time early time period will be sensitive to differences between stuttering and non-stuttering populations (see discussion of Saltuklaroglu et al., 2017 in “Auditory Discrimination Tasks” section).

This rich source of combined excitatory and inhibitory information measured across time is not available *via* other techniques such as fMRI due to poorer temporal resolution and the inability to distinguish spatially co-located predictive and inhibitory processes. This is because the balance of neural activity within a given patch of the cortex (i.e., voxel) is governed by both excitatory and inhibitory processes, leading to increases and decreases of cerebral blood flow, respectively (Devor et al., 2007; Goense et al., 2012). Hemodynamic signals related to opposite changes within a given voxel may cancel each other out, with the observed hemodynamic response reflecting the difference between co-localized excitatory and inhibitory processes rather than absolute measures of excitation and inhibition (Xu, 2015).

Alongside attention, working memory function is critical to the successful completion of cognitive tasks as perceived stimuli are retained for task-related processing. Neural correlates of working memory can be clearly observed in event-related EEG data (Schneider et al., 2017; Jenson et al., 2019). Many lines of research have demonstrated strong post-stimulus strong alpha and beta desynchronization following stimulus offset, which is interpreted as the processing of stimuli while held in working memory. This also is the most consistent finding in our studies that require participants to make same/different judgments regarding pairs of auditory stimuli (Bowers et al., 2013; Jenson et al., 2014; Thornton et al., 2018, 2019). It is interesting to note that this is the same pattern that is observed in overt and covert speech production (Jenson et al., 2014), suggesting that at some level, perceived acoustic information is being covertly replayed as is retained in working memory. It is also particularly interesting that retention of information within PWM may operate *via* the instantiation of the same forward and inverse modeling mechanisms that drive overt speech (Alho et al., 2012; Pickering and Garrod, 2013) and may be compromised in PWS. Saltuklaroglu et al. (2017) (see “Time-Frequency Differences” section for more in-depth discussion) did not find post-stimulus mu rhythm oscillatory differences between PWS and TFS in an auditory discrimination task. However, they did not employ a task that was designed to load working memory. In contrast, new findings from the Bowers lab (see “Design and Hypotheses of the Preliminary Study” section for a more detailed discussion) employ tasks designed to load working memory for nonword syllable sequences that have previously been associated with differences in behavioral accuracy between PWS and TFS and are revealing mu oscillatory differences during the task.

Based on the descriptions above, there are at least three compelling reasons why we argue that measurements of mu rhythms can shed much-needed light on the neurophysiology of stuttering: (1) the anterior sensorimotor regions over which they are recorded integrate input from both internal modeling and basal ganglia loops; (2) mu-alpha and mu-beta can capture distinct contributions from sensorimotor feedback and forward modeling (respectively) with basal ganglia influences over the time course of an event; and (3) the ability to capture patterns of both synchronization and desynchronization of neuronal populations provides valuable measures of inhibition and activation that illuminates precisely how neural activity changes within a single event. This stands in contrast to measures that simply average neural activity across an event, possibly without a means of capturing inhibitory contributions. To this end, we will briefly summarize two of our published studies showing differences between matched PWS and TFS in fluent speech production and in auditory discrimination tasks. We will then present some new data showing group differences in a repetition task that recruits working memory processes.

## Speech Production

**Figure 2:**
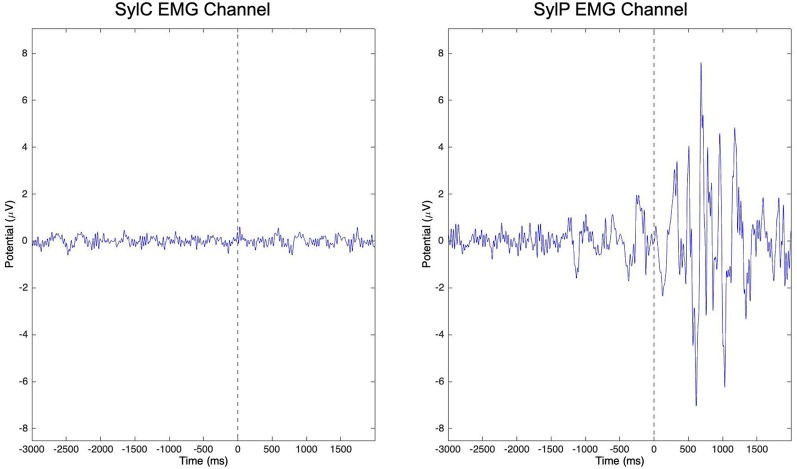
Peri-labial electromyographic (EMG) channel data from all subjects in covert (SylC) and overt (SylP) syllable production following a visual inspection. The vertical dashed line in each graph represents the cue to initiate production. While peri-labial EMG activity in SylP is characterized by preparatory activity prior to the cue to speak followed by robust activity following the speech cue, minimal peri-labial activity is observed over the time course of SylC. Data has been adapted from Jenson et al. (2018).

A number of studies have identified mu oscillatory activity during speech production (Jenson et al., 2014; Mandel et al., 2016; Kittilstved et al., 2018), and the sensitivity of mu oscillations to internal modeling processes makes them well-suited to interrogate notions of compromised sensorimotor processing in PWS. However, in order to better understand how underlying sensorimotor function for speech differs in PWS relative to TFS, it is necessary to compare EEG recordings from spontaneously fluent speech that is free from overt stuttering or therapeutic fluency enhancing strategies. Jenson et al. (2018) capitalized on the abilities of PWS to produce spontaneously fluent simple utterances and the temporal precision of EEG to compare mu oscillatory activity from PWS and TFS during covert (i.e., imagined) and overt production of orthographically presented syllable pairs and words. To ensure that subjects refrained from movement during covert production trials, raw channel data were visually inspected, and any trials in which the peri-labial electromyographic (EMG) channel demonstrated large deflections from baseline were excluded from further analysis. The raw EMG channel data from covert and overt syllable production trials is shown in Figure 2 to demonstrate the effectiveness of visual inspection for exclusion of trials containing movement. Neural data from stuttered trials were also excluded from the analysis. Using ICA, Jenson et al. (2018) were able to identify mu components and peri-labial EMG components. Time-frequency decompositions of the EMG component confirmed that the two groups were behaviorally equivalent on speech tasks with respect to timing and strength of muscle activity. The EMG data could then be mapped temporally to the neural data, which revealed a number of group differences (discussed in “Overt Speech Differences” to “Right Hemisphere Comparisons” sections below).

### Overt Speech Differences

**Figure 3:**
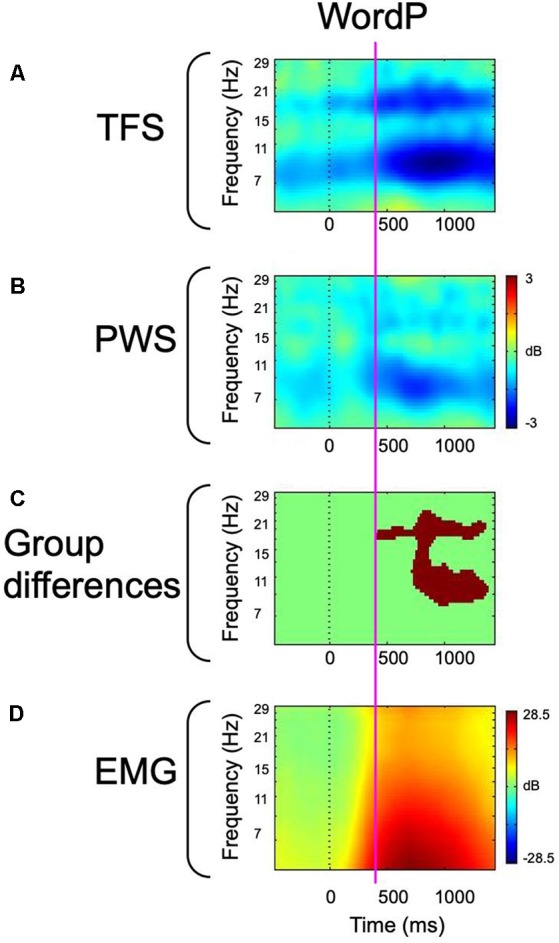
Event-related spectral perturbation (ERSP)-decomposed left hemisphere mu and peri-labial EMG data from overt word production. The vertical dotted line represents the cue to initiate production. **(A)** ERSP data from fluent controls. **(B)** ERSP data from participants who stutter. **(C)** Between-group statistical comparisons with cluster corrections for multiple comparisons. Red voxels are significant at *p* < 0.05 (corrected). **(D)** Peri-labial EMG activity. The vertical magenta line illustrates the temporal concordance between the emergence of robust alpha and beta desynchronization, statistical differences, and the onset of peak EMG activity. Data has been adapted from Jenson et al. (2018).

In overt production conditions, both PWS and TFS produced weak left hemisphere mu-alpha and mu-beta desynchronization prior to the cue to initiate production, with robust desynchronization emerging following the cue to produce speech and temporally aligned with the onset of peak EMG activity (Figure 3). The presence of weak mu-alpha and mu-beta desynchronization during orthographic stimulus presentation was interpreted as evidence of the speech network setting up (Gehrig et al., 2012), such that participants were ready to initiate production when cued. During word production, PWS produced weaker mu-alpha and mu-beta desynchronization across the time course of speech production, which were interpreted within the framework of State Feedback Control (Houde and Nagarajan, 2011) as evidence of reduced internal modeling activity in line with the proposals of Max et al. (2004). Specifically, reduced mu-beta desynchronization was interpreted as evidence of weak forward modeling, while reduced mu-alpha desynchronization was interpreted as evidence of reduced evaluation of sensory feedback. This interpretation was supported by the lack of differences in either the strength or timing of peri-labial EMG in overt production conditions. As the strength and timing of movement parameters are encoded in sensorimotor oscillations (Korik et al., 2018; Li et al., 2018), primary motor and somatosensory (i.e., external loop) influences on mu activity cannot account for observed group differences, and we propose that they represent differential internal modeling activity within the internal loop. As these differences were present in spontaneously fluent speech, we suggest that they represent an underlying sensorimotor instability that predisposes the speech of PWS to breakdown. However, in order to more fully interrogate internal loop dynamics in PWS, it remains critical to examine sensorimotor activity arising from covert speech tasks, in which external loop activity is not elicited.

### Covert Speech Differences

**Figure 4:**
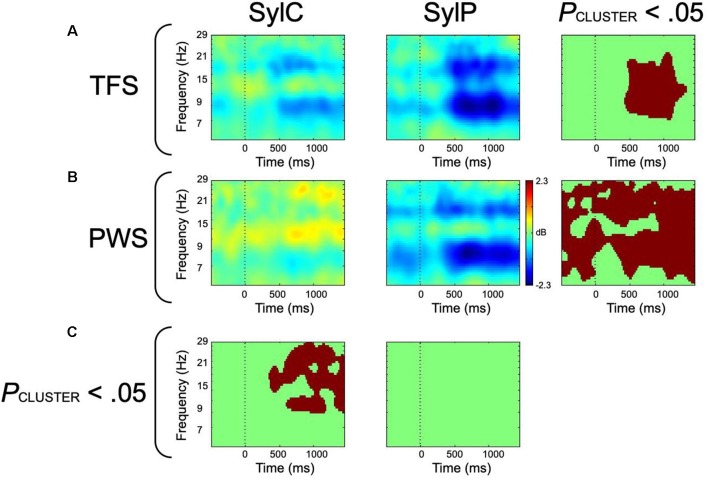
ERSP-decomposed left hemisphere mu data from covert (SylC) and overt (SylP) syllable production. The vertical dotted line represents the cue to initiate production. **(A)** ERSP data from fluent controls, with the right-most column representing within-group differences. **(B)** ERSP data from participants who stutter, with the right-most column displaying within-group differences. **(C)** Between-group differences. All statistical comparisons employed cluster corrections for multiple comparisons, and red voxels represent significant differences at *p* < 0.05 (corrected). Data has been adapted from Jenson et al. (2018).

In covert syllable production, patterns of mu activity were similar, yet weaker than those observed during overt production (Figure 4). This is consistent with the notion that the influence of sensorimotor and primary motor/somatosensory responses on mu oscillations are additive in nature. As covert speech is supported by internal modeling processes (Tian and Poeppel, 2010, 2012; Tian and Poeppel, 2013), without the primary motor/somatosensory responses elicited during covert movement tasks, differences are interpreted as being solely related to sensorimotor function. Weaker mu-alpha and mu-beta desynchronization were observed in PWS compared to TFS, paralleling the group differences observed during overt word production. Reduced mu-beta desynchronization in PWS was interpreted as evidence of reduced forward modeling consequent to a trait-related sensorimotor deficit. Reduced mu-alpha desynchronization in PWS was interpreted to suggest that in the absence of reafference, sensory feedback estimation *via* the internal loop is compromised. This inability to internally estimate sensory feedback thus exacerbates compromises to forward modeling.

An inability for PWS to estimate sensorimotor feedback through the internal loop is corroborated by within-group differences between covert and overt syllable production. In TFS increased mu-alpha and mu-beta desynchronization was noted from ~400 to 1,200 ms following the cue to produce speech, which aligned with the time course of peak EMG activity (Figure 4). As this increased activity in the presence of a movement requirement is restricted to the time course of movement, it likely reflects the contributions of the primary motor (beta) and somatosensory (alpha) responses on mu activity. In contrast, PWS demonstrated significantly increased mu-alpha/beta desynchronization prior to and throughout speech production, suggesting that this increased mu activity reflects more than the additive effect of primary motor and somatosensory responses. It may be proposed that the presence (or potentially even the anticipation) of reafference primes the sensorimotor system in PWS, compensating for underlying sensorimotor deficits and enabling internal modeling activity in the compromised left hemisphere. This increased mu activity in PWS in the presence of a movement requirement may mask the underlying sensorimotor deficits observed during covert syllable production, accounting for the lack of group differences during overt syllable production.

### Right Hemisphere Comparisons

In contrast to the robust differences observed in the left hemisphere, no group differences observed in the right hemisphere mu activity in any condition. This finding was unexpected given existing notions of right hemisphere compensation for a compromised left hemisphere sensorimotor mechanism (Preibisch et al., 2003; Neumann et al., 2005; Kell et al., 2009). While these findings may appear to undermine notions of right hemisphere compensation in PWS, it is critical to consider the relative contributions of right and left hemispheres to sensorimotor processing for speech. In TFS, right hemisphere patterns were similar, yet weaker than those observed in the left hemisphere, consistent with reports that sensorimotor transformations for speech are bilateral (Cogan et al., 2014) yet left hemisphere dominant (Hickok and Poeppel, 2007). However, the lack of such a hemispheric decrement in PWS suggests that the contribution of the right hemisphere is proportionally larger in PWS. Consistent with notions that PWS are overly reliant on sensory feedback (Max et al., 2004) and reports that corrective feedback signals are mediated by the right hemisphere (Tourville and Guenther, 2011), these findings suggest a proportionally larger contribution of reafference to speech motor control in PWS. However, more work is necessary to clarify differential hemispheric contributions to sensorimotor control for speech in PWS.

### Interpretation

The significantly reduced mu-alpha and mu-beta desynchronization across the time course of spontaneously fluent overt speech and covert speech production suggests that even the fluent speech of PWS is characterized by differential sensorimotor activity. This underlying sensorimotor deficit makes the speech of PWS characteristically unstable and prone to breakdown. However, several questions remain to be addressed. First, it is not apparent why, if these sensorimotor deficits are present in even the fluent speech of PWS, speech disruptions are only intermittently present. Second, it remains unclear how these findings relate to the results of Mersov et al. (2016), who reported elevated mu-beta desynchronization prior to speech in PWS, interpreting it as a stronger facilitatory signal needed to disinhibit a more strongly inhibited motor system. Third, as internal modeling processes are active across a number of perceptual, cognitive, and motor-based processes, it remains unclear why the behavioral characteristics of stuttering are restricted to speech production. Future work is necessary to clarify these and other questions regarding sensorimotor influences on stuttering.

## Auditory Discrimination Tasks

Many studies have evaluated sensorimotor activity during speech and tone discrimination tasks. Activity is typically heightened in more difficult listening conditions, such as in the presence of noise. Therefore, in order to observe sensorimotor activity in a cognitive task that eliminates mu activity related to movement (and possibly stuttering) Saltuklaroglu et al. (2017) compared mu rhythm spectra and oscillatory activity in a control condition (passively listening to white noise at 70 dB SPL) and four auditory discrimination conditions. The discrimination conditions required participants to make same/different judgments of either syllable or tone pairs in either quiet or noisy (+4 dB SNR) backgrounds. Group differences were found both in mu spectra and event-related oscillatory power (discussed in “Spectral Differences” and “Time-Frequency Differences” sections below).

### Spectral Differences

**Figure 5:**
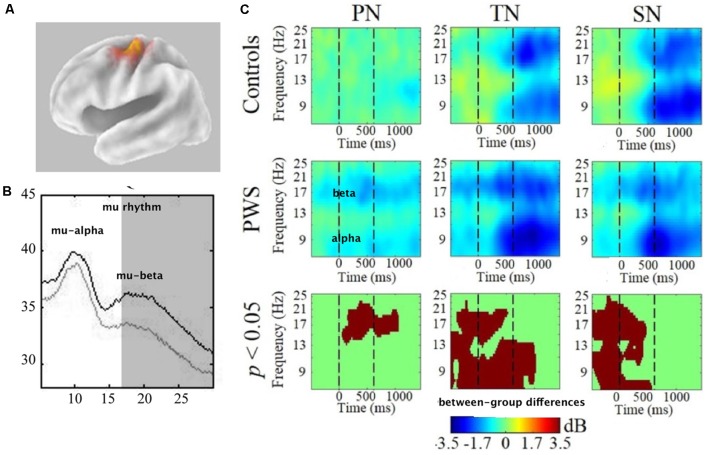
**(A)** Van Essen image average template of left mu source (localized to BA6-premotor cortex). **(B)** Comparison of mu spectra for one condition (TN—discriminating tones in noise) showing significant differences in mu-beta (shaded) spectral amplitudes. All conditions showed this difference bilaterally. **(C)** Time-frequency decompositions of mu-alpha and mu-beta relative to baseline, showing significant group differences in PN (passive noise), TN and SN (discriminating syllables in noise). For TN and SN, stimuli were presented from time = 0–600 ms. Therefore, pre-stimulus attention is measured prior to 0 ms and post-stimulus working memory is measured after 600 ms. Warmer colors (e.g., yellow) depicts event-related synchronization (ERS) and cooler colors (e.g., blue) depict event-related-desynchronization (ERD). Data has been adapted from Saltuklaroglu et al. (2017).

PWS displayed mu spectra with lower mu-beta amplitudes bilaterally across the control condition and all experimental conditions (Figure 5B). In other words, mu-beta spectral peaks were reduced in PWS regardless of the task, stimuli, or the presence of noise. Considering that mu-beta rhythms are thought to encode forward models, these findings appear to be consistent with stuttering being related to weak or unstable forward modeling (Max et al., 2004). The findings raise the possibility that reduced mu-beta amplitude might be a neural biomarker for stuttering. However, as data were recorded from an adult cohort, it must be considered that observed spectral differences may be influenced by cortical reorganization due to a lifetime of stuttering (Doyon and Benali, 2005; Dayan and Cohen, 2011). To bolster notions of reduced mu-beta amplitude constituting a biomarker for stuttering, it is necessary to test children who stutter close to the age of onset to minimize the potential for neuroplastic change secondary to a prolonged period of stuttering. Additionally, it is necessary to evaluate mu-beta power in resting-state tasks in which the spectra are least influenced by oscillatory activity related to cognition or movement. While future work is required to validate notions of a spectral biomarker for stuttering, this paradigm holds promise for identifying children at risk, raising the tantalizing possibility of early intervention prior to the onset of stuttering.

### Time-Frequency Differences

In contrast to the spectral data, only the conditions that involved background noise produced group differences in the time-frequency decomposition analysis (Figure 5C). These differences were only found in the left hemisphere. The most surprising finding was that PWS demonstrated bilateral mu-beta desynchronization in the control condition that only entailed passively listening to noise. As beta desynchronization is considered a motor response, this finding suggests that the introduction of task-irrelevant noise is sufficient to elicit motor activity in PWS. A number of questions arise from this finding. Does background noise impact speech fluency? The presence of high-intensity masking noise sufficient to drown out acoustic reafference from speech has been demonstrated to enhance fluency for some PWS (Block et al., 1996; Fiorin et al., 2019). However, the levels presented in this study were only 70 dB and no speech production tasks were included in the experiment. Thus, questions remain regarding the impact of lower levels of background noise on speech fluency.

In the noisy discrimination conditions, patterns of mu-alpha and mu-beta oscillations relative to baseline can be observed across the time course of discrimination events. While both groups displayed mu oscillatory activity consistent with processing and retaining auditory stimuli in working memory, differences were observed in the early attentional segment of the event. TFS displayed patterns of mu-alpha synchronization that have been observed in similar studies. As it was only observed in the noisy conditions, it was interpreted as task-related inhibition that functions to suppress irrelevant information (i.e., background noise). This early inhibitory activity was significantly reduced in PWS in the left hemisphere (Figure 5A), a finding that appears to be consistent with reports of reduced auditory gating (Kikuchi et al., 2011) in basal ganglia based inhibitory mechanisms of PWS (Civier et al., 2010). Importantly, however, the presence of background noise did not impact the PWS ability to discriminate any more than TFS. Thus, additional questions arise regarding the impact of noise on cognitive function in PWS. With reduced inhibitory function, are PWS able to compensate for background noise in other ways? Will higher levels of background noise produce significant reductions in discrimination abilities? Do PWS have a lower tolerance for background noise?

## Phonological Working Memory

### Background and Need for Preliminary Data

In addition to speech production and auditory speech processing, the mu rhythm may also be useful for examining how sensorimotor cortex rhythms are related to maintaining phonological representations in working memory and in executing a sequence of speech sounds from working memory. A commonly used task for investigating PWM is a nonword repetition task in which a given subject is required to listen to a sequence of speech sounds that conforms to phonological rules in the language but has no lexical representation or semantic content (Baddeley, 2012). The task requires listening to and encoding the sounds, holding them in working memory, and then reproducing the sounds in the order in which they were presented. A growing body of evidence implicates load-dependent differences in nonword repetition in both children (CWS) and adults (AWS) who stutter compared to matched controls (Bowers et al., 2018; Ofoe et al., 2018). Recent studies investigating nonword repetition tasks have demonstrated overall lower performance in both preschool CWS (Spencer and Weber-Fox, 2014; Pelczarski and Yaruss, 2016) and AWS (Byrd et al., 2012, 2015, 2017, 2018; Coalson and Byrd, 2017).

In preschool CWS, the available evidence suggests that differences in nonword repetition are subclinical and load-dependent and in adults differences are apparent only under high loads at the limit of typical capacity to hold speech sounds in memory (e.g., 7 syllable nonwords) or under other syllable stress-related load manipulations (Bowers et al., 2018). Further, nonword repetition has been reported to differentiate preschool CWS who persist from those who recover, suggesting that the underlying cognitive capacities supporting PWM may be a marker for the phenotypic expression of recovery or persistence among other cognitive-linguistic capacities (e.g., syntax; Spencer and Weber-Fox, 2014; Usler and Weber-Fox, 2015). Despite its potential significance as a simple measure that could be useful both in a clinical setting and as a construct in theoretical frameworks, one barrier to understanding why CWS and AWS are different on the task has been separating various cognitive and corresponding neurophysiological processes associated with task performance and behavioral accuracy (Bowers et al., 2018).

Nonword repetition tasks require at least sustained attention to speech sounds over a number of trials, the capacity to hold the speech sounds for up to a few seconds, and then to accurately execute the sequence in the order it was presented. Further, both CWS and AWS present with subtle differences in a number of cognitive capacities that could affect behavioral performance on a nonword repetition task, including attention/executive function (Postma and Kolk, 1993; Alm, 2004; Eggers et al., 2012, 2013; Eggers and Jansson-Verkasalo, 2017), phonological encoding (Postma and Kolk, 1993), speech planning (Howell and Au-Yeung, 2002), speech-sound processing (Neef et al., 2012; Saltuklaroglu et al., 2017), and differences in speech-motor control for execution interacting with cognitive and emotional factors (Namasivayam and van Lieshout, 2011; Smith and Weber, 2017). In addition, it is also unclear how factors such as working memory load (e.g., number of syllables) contribute to observed differences in previous studies (Pelczarski and Yaruss, 2016).

It is possible that any one of these processes or an amalgam accounts for the difference in behavioral performance and it is unclear from behavioral studies alone what processes account for differences in speech-motor output (Spencer and Weber-Fox, 2014). As an example, speech-motor output, measured as incoordination in speech articulators (i.e., lip aperture variability), differs significantly in a nonword repetition tasks even when no behavioral differences are observed at lower loads (e.g., 4 syllable repetition), suggesting that while motor control differs in AWS and CWS it may be distinct from behavioral accuracy (Smith et al., 2010, 2012). As such, it will be critical in the future to enhance understanding of what cognitive and sensorimotor processes are related to or mediate differences in nonword repetition performance and in turn the mechanistic processes underlying differences in PWM (Bowers et al., 2018).

A number of neurophysiological frameworks propose that prefrontal, premotor and sensorimotor systems mediate short-term phonological storage in coordination with temporal and temporal-parietal regions critical for sensorimotor integration in speech production (Hickok and Poeppel, 2007; McGettigan et al., 2011; Herman et al., 2013; Majerus, 2013). In particular, a network of regions known as the dorsal stream may play a critical role in mapping acoustic speech sound representations to the motor commands required to produce them as children learn new lexical representations and to produce speech-sounds in the context of words (Hickok et al., 2011). Neuroimaging studies have demonstrated that the dorsal stream is active in tasks requiring the repetition of sequences of speech sounds after a delay period, suggesting that sensorimotor integration in the dorsal stream may play an important functional role in PWM (Hickok et al., 2003; Markiewicz and Bohland, 2016; Perrachione et al., 2017). Recent studies using MEG and EEG have provided evidence that timely coordination between dorsal stream premotor and the parieto-temporal regions during the maintenance of syllable sequences is related to repetition performance and processing load (Herman et al., 2013). The timely coordination between cortical rhythms in the premotor cortex and parieto-temporal junction, in particular, may be critical for the accurate reproduction (i.e., motoric execution) of syllable sequences from working memory over the sensorimotor cortex. In other words, high time-resolution approaches suggest that coordination between premotor, posterior sensory and motor cortices bilaterally at different phases of the task may be required both for maintaining syllable sequences in working memory and for accurately executing them when a response is required (Majerus, 2013). For that reason, high time resolution approaches have the potential to shed light on what processes are different in PWS as they perform various phases of the task.

Current theoretical frameworks designed to account for recent neuroimaging findings in CWS have also suggested that stuttering may arise from subtle differences in the coordination of large-scale cortical-subcortical networks central to which is a deficit in coordinative sensorimotor timing (Chang et al., 2019). Thus, a timing deficit related to the sensorimotor control of speech has the potential to account for load-dependent differences in tasks loading PWM and in speech output in more naturalistic conditions (Bowers et al., 2018). Recent EEG studies of neural oscillations using word and nonword repetition tasks suggest that power and measures of inter-electrode coordination (i.e., phase coherence) are related to stuttered or fluent speech production trials (Sengupta et al., 2017). However, no studies have used a time-sensitive approach, like those used in previous studies of auditory speech processing and production (Saltuklaroglu et al., 2017; Jenson et al., 2018), to examine at what phase of the task differences in sensorimotor processes emerge under high and low PWM loads. Because it is involved in sensorimotor integration in both speech processing and production tasks, an examination of the mu rhythm in a nonword repetition task in AWS and TFS may provide a place to start investigating at what phase of the task sensorimotor integration processes differ in AWS compared to TFS.

### Design and Hypotheses of the Preliminary Study

**Figure 6:**
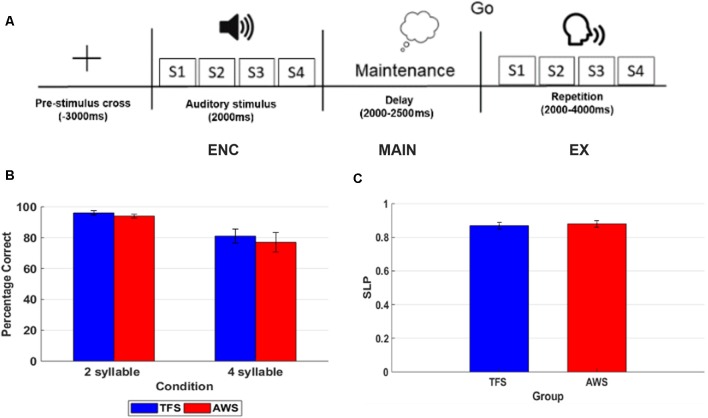
This figure shows an example of a timeline for one trial in the 4 syllable repetition condition with each phase of the task labeled and behavioral accuracy on the 2 and 4 syllable tasks in the typically fluent speaker (TFS) and adults who stutter (AWS) groups. **(A)** Timeline of one 4 syllable trial. **(B)** Percentage correct trials in the 2 syllable and 4 syllable condition with TFS shown in blue and the AWS group depicted in red. **(C)** Syllable load performance metric (SLP) in the TFS (blue) and AWS groups (red).

To determine the feasibility of measuring processing differences in the sensorimotor mu rhythm, a simple syllable sequence reproduction task was employed to examine group differences between age and sex-matched TFS and AWS. The syllable repetition task was selected to minimize lexical and syntactic influences that can occur in nonword repetition paradigms (Herman et al., 2013). In the task, 11 TFS and 11 AWS were asked to simply listen to two or four bilabial, consonant-vowel (CV) syllables and repeat the sequence following a short delay cued with a visual image. To successfully complete the task for each trial, participants must listen to the syllables (encoding), maintain the syllable sequence in working memory over a short delay period (maintenance), and then execute the sequence (execution). A sample timeline for the task is displayed in Figure 6. Based on previous behavioral studies, we hypothesized that, while the task would manipulate load (i.e., 2 vs. 4 syllable differences), it would not result in differences in behavioral performance between the two groups (Bowers et al., 2018). The rationale for using two relatively low load conditions was to control for behavioral performance (i.e., similar performance across groups) while evaluating differences in neural processing between the two groups. Based on previous studies of speech processing and production in the mu rhythm, we expected lower suppression during a covert rehearsal period to hold sound sequences in working memory and significantly lower suppression during execution in AWS compared to TFS.

### Methods

#### Participants

Prior to participation in the study, participants in both groups provided written, informed consent approved by the institutional review board at Idaho State University and at the University of Arkansas. To determine the degree of handedness, the Oldfield Handedness Inventory was administered to all participants in the study. Eleven AWS (three females) scoring in the range of “usually” or “always” right-handed were recruited from Idaho State University and surrounding regions and were age and sex-matched with 11 TFS. The demographic characteristics of matched pairs are shown in Table 2. The AWS was diagnosed by a licensed speech-language pathologist with more than 5 years of experience evaluating stuttering. As a part of the initial evaluation, the Stuttering Severity Instrument 4th edition was administered to determine the current severity of stuttering. Participants ranged from moderate to severe. In addition, the Overall Assessment of the Speaker’s Experience of Stuttering (OASES) was given to nine of the participants to determine both the current severity of stuttering and the speakers’ attitudes toward stuttering (Yaruss and Quesal, 2006). The AWS reported no cognitive, neurological injury, or other attentional disorders apart from developmental stuttering. Eleven age and sex-matched typically fluent controls were also recruited from Idaho State University and surrounding region and were matched pairwise with AWS. TFS participants were also scored in the “usually” or “always” right-handed range and reported no history of neurological, cognitive, or attentional disorders. All participants provided written, informed consent prior to participation in the study approved by the Idaho State University and University of Arkansas institutional review boards.

**Table 2:** Demographics of age and gender matched pairs of adults who stutter (AWS) and typically fluent speaker (TFS) participating in phonological working memory study.

Subject ID	Age	Sex	Subject ID	Age	Sex
AWS1	55	M	TFS1	54	M
AWS2	46	M	TFS2	47	M
AWS3	18	M	TFS3	19	M
AWS4	24	F	TFS4	26	F
AWS5	35	M	TFS5	34	M
AWS6	24	F	TFS6	22	F
AWS7	35	M	TFS7	39	M
AWS8	24	M	TFS8	23	M
AWS9	19	M	TFS9	20	M
AWS10	33	M	TFS10	35	M
AWS11	26	F	TFS11	22	F

#### Stimuli

Syllable stimuli were generated by an adult male speaker and were recorded using PRAAT software on a Dell 2.7 Ghz desktop computer. Recordings consisted of the syllables /ba/, /ma/, /pa/, and /wa/. Syllable sequences were normalized to have the same root-mean-square amplitude using PRAAT and were 430 ms on average with a 60 ms interstimulus interval between syllable presentations. Syllable sequences were constructed such that within 2 syllable trials no syllable was repeated and within 4 syllable trials no pair was repeated. Trials were presented in two blocks of 40 trials each with a 5-min rest period offered between blocks. The 2 and 4 syllable trials were presented in random order using E-Prime software. Acoustic stimuli were presented at a comfortable loudness level (~70 dB) *via* Eytmotic ER-1 occluding ear insert headphones. Visual stimuli (cross and go cue) were presented on a 15-inch monitor placed 132 cm from the participant.

#### Procedure

The experiment was conducted in an electrically and magnetically shielded sound-attenuated room. Participants were seated in a comfortable chair with their heads and necks well supported. Stimuli were presented using a 2.7 GHz Dell computer *via* E-Prime, version 3.0. Timing of responses and events were verified independently using a timing device manufactured by Electrical Geodesics. Participants were instructed to listen to the syllables and wait to repeat the syllables when a visual cue (a drawing of speaking head) appeared on the monitor. Prior to the experimental conditions, the participants were required to complete five practice trials that were not included in the analysis. The entire experimental session was recorded audio-visually *via* a camera situated just in front of the participant for later manual judgments of both stuttering in trials and the accuracy of repetition. A trained speech-pathology graduate assistant and a certified speech-language pathologist with more than 5 years of experience in stuttering coded the trials as stuttered or fluent. Trials were judged as stuttered if a trained rater observed a part-word repetition, prolongation, or articulatory block (Riley, 1972; Riley and Bakker, 2009). All trials that were stuttered (fewer than 1%) were rejected from the analysis. To determine interrater reliability, a Cohen’s κ value of 0.90 was obtained between the two raters. Trials were manually judged as correct if the participants responded within 3,000 ms following the response cue and produced the complete sequence in the same order as the target sequence. Measures of behavioral accuracy included both a measure of % correct trials out of the trials submitted and a measure of load adapted from a previous study known as syllable load performance (SLP; Herman et al., 2013). SLP is a measure of processing load that accounts for relative performance on the two tasks in each individual to derive a measure of load processing across the tasks.

#### EEG Data Acquisition and Processing

A 128 channel Electrical Geodesics recording system was used to obtain EEG data during the tasks and in a 5 min, eyes-open resting-state baseline. The EMG signal was recorded from a single bipolar channel placed above and below the lips using an integrated Physio 16 system (Jenson et al., 2015; Bowers et al., 2018). Blood pressure was also monitored using infrared sensors placed on the index finger. Procedures for fitting and preparing the nets were followed in accordance with previous studies and recommendations from Electrical Geodesics, including head measurements, electrolyte preparation, and net placement on each subject’s scalp (Ferree et al., 2001; Song et al., 2015). Impedances were never greater than 50 kΩ as examined prior to and following a study session (Ferree et al., 2001; Dalla Volta et al., 2018). Data were processed using the same steps in studies of speech processing and production described in sections “Speech Production” and “Auditory Discrimination Tasks” including data pre-processing, the application of ICA, and time-locking to stimulus events (i.e., epoching) prior to analysis of event-related spectral perturbations (ERSPs; Bowers et al., 2013; Jenson et al., 2015).

First, EEG data were bandpass filtered from 1 to 70 Hz using a zero-phase, finite impulse response (FIR). The FIR is windowed sinc filter using a Hamming window, with a transition bandwidth of 1 Hz, and cutoff frequencies between 0.5–70.5 Hz. The filter uses a heuristic for determining transition bandwidth that is 25% of the passband edge and distance from the passband edge to the critical frequency. EEG data were then downsampled to 256 Hz from the original sampling rate of 1,000 Hz and referenced using the average common reference including all scalp-channels (i.e., excluding extraocular and EMG channels). The continuous data prior to epoching were denoised using visual inspection for gross one-time artifact known to affect ICA decomposition (Delorme et al., 2012) and subsequently, artifact subspace reconstruction (ASR) was used to remove channels with excessive spatial drift and spectral characteristics associated with line noise or other non-repetitive myographic artifacts (Jenson et al., 2014; Bowers et al., 2018). CleanLine was also used to reduce the remaining line noise visible in the spectrum between 40 and 70 Hz (Leske and Dalal, 2019). The mean number of rejected trials was 11 across conditions. The data were epoched around the time-locking event (i.e., acoustic syllable presentation) from −1,000 ms prior to the event to 9,000 ms following the event. Subsequently, ICA using the *binica* algorithm was applied with a principle component reduction to the number of channels not exceeding ASR thresholds (Chang et al., 2019). No more than eight channels were rejected (mean 5) out of the original 128. Following ICA, dipole fitting was applied using the Dipfit toolbox as in other studies reviewed in sections “Mu-alpha and Mu-beta in Movement and Cognitive Tasks” and “Covert Speech Differences” (Bowers et al., 2013, 2014, 2019; Jenson et al., 2014, 2015; Cuellar et al., 2016; Saltuklaroglu et al., 2018a). The multiple artifact rejection algorithms (MARA) was used to identify and remove components identified as an artifact with a probability greater than 0.40 (Winkler et al., 2014). Finally, component spectra, ERSPs, and intertrial phase coherences were computed for each independent component (IC) that could be a fit with a single dipole with less than 20% residual variance. Only those ICs that could be a fit with a single dipole were retained. Principle component clustering was used to cluster components across participants and groups using ERSPs, spectra, intertrial coherence measures (Delorme et al., 2011). The threshold for rejecting outlier components was 3 SD from any cluster mean. Within and between-subject differences in the 2 and 4 syllable conditions were examined using a permutation test with a cluster correction for multiple comparisons across the time-frequency matrix (117 × 200). As in our previous studies, a nonparametric permutation test was used because time-frequency values are not normally distributed. As each processing stage has the potential to introduce artifact, a subset of the data was reprocessed with a milder processing pipeline, yielding similar results. The presence of similar, albeit noisier, results when a different set of pre-processing steps were employed highlights both the robust nature of the observed effects and benefit of the full processing pipeline.

### Results

**Figure 7:**
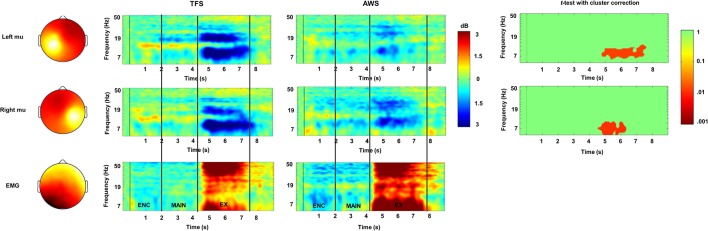
ERSPs in the encoding (ENC), maintenance, (MAIN) and execution (EX) phases of the 4 syllable repetition task in TFSs (rows) and in AWS (columns). Mu rhythm scalp-topographies and a cluster associated with peri-labial EMG during execution are shown to the left with scalp-potential distributions for the component cluster with white-yellow showing greater density and red showing lower density. ERSPs are depicted in time-frequency scalograms with frequency on the y-axis and time on the x-axis. Significant group differences are shown in the third column (cluster corrected *t*-test) with *p*-values <0.05 (range 0.05–0.01) shown in red and non-significant values shown in green.

Mean % correct repetition trials in the 2 and 4 syllable tasks and SLP for the AWS and TFS groups are shown in Figure 7. Behavioral results showed that both groups were less accurate in the 4 syllable compared to the 2 syllable task. A repeated-measures analysis of variance (ANOVA) with the factors group and condition showed an effect of condition but no significant effect for the group. As in previous studies using syllable perception and production tasks, a network of IC clusters was identified, including the frontal lobe, temporal lobe, parietal lobe, and occipital lobe IC clusters. As in earlier studies, the mean % RV across clusters was <6% and the mean for the left mu was 5.95% and for the right 5.35%, suggesting a single dipole model adequately accounted for IC sources within the head model. Dipole locations ranged from the precentral to postcentral gyrus and were most dense over the precentral gyrus in both the left and right hemispheres. Eight participants from the AWS group and eight matched subjects from the TFS group contributed mu components to the left and right mu clusters. Visual inspection of individual and mean ERSPs across trials showed mu-alpha/beta desynchronization relative to the silent intertrial interval in both the maintenance time period and during the execution time period across both the AWS and TFS groups while synchronization (i.e., increases in power) were observed during the listening time period.

Mean ERSPs in the encoding, maintenance, and execution phases of the task for both groups in the high load condition (i.e., 4 syllable condition) are shown in Figure 7. Permutation statistics adopting a cluster correction for multiple comparisons across the entire time-frequency matrix (117 × 200) were used to evaluate group and condition differences. The within-subjects comparison showed no significant differences between the 2 and 4 syllable conditions. A comparison of the group on the 2 syllable and 4 syllable conditions showed significantly lower desynchronization in the mu-alpha band in the AWS group that was restricted to the execution period in both the low load 2 syllable condition and the 4 syllable condition. There was no significant interaction for the factors (Group) and (Condition). Although not significant, there was a trend toward lower desynchronization in the mu-beta band in the AWS group compared to the TFS group. A subsequent Pearson bivariate correlation showed a trend toward a mild relationship with no significant correlation between mu-alpha desynchronization and individual behavioral performance. Overall, the results implicate reduced mu-alpha suppression in the left and right sensorimotor mu rhythm during speech execution with a trend toward lower left hemisphere desynchronization in the mu-beta band.

### Implications

The purpose of this preliminary investigation was to examine at what phase of a syllable repetition task differences in mu rhythm desynchronization emerged between AWS and TFS. We hypothesized group differences in mu-alpha/beta desynchronization between AWS and TFS in the maintenance and execution periods of a syllable repetition task. Preliminary findings showed a significant difference in the left and right sensorimotor mu rhythms primarily during execution, with a trend toward differences in the mu-beta band during the maintenance phase of the task in the left component cluster. As in previous studies of covert syllable production, mu-alpha and mu-beta desynchronization relative to the baseline occurred during the maintenance period, suggesting that bilateral mu rhythms were engaged in covert rehearsal of the stimuli prior to overt execution. A preliminary correlation analysis did not provide strong support for a relationship between differences in mu-alpha desynchronization and behavioral accuracy. Findings suggest that observed bilateral group differences in mu activity are related primarily to motoric execution as opposed to maintenance in working memory or syllable encoding processes. As such, results suggest that a time-sensitive signal separation approach previously applied to speech perception and production tasks may also aid in identifying separable physiological processes related to syllable repetition tasks in AWS.

While a growing body of evidence implicates lower accuracy in preschool CWS and AWS compared to TFS on nonword repetition tasks (Bowers et al., 2018; Ofoe et al., 2018), the current challenge is to identify the cognitive processes related to differences in accuracy. Because nonword repetition tasks load a number of cognitive and sensorimotor processes over the course of the task, it is unclear which of these processes may be different in CWS and AWS. Such information is critical to determining which processes account for differences in behavioral accuracy and thus may be important for identifying which of the task demands in nonword repetition are related to subsequent recovery or persistence in pre-school children. These preliminary results suggest one process that differs between the groups is in the sensorimotor (i.e., motor and somatosensory) processing related primarily to the execution of syllable sequences. Those findings are broadly consistent with studies showing differences in inter-articulator coordination even in the absence of behavioral differences at lower nonword repetition loads, suggesting that cortical differences in processing are likely to be related to subtle differences in peripheral execution (Smith et al., 2010, 2012). Further, the analysis implies that lower mu-alpha and mu-beta desynchronization primarily during execution is separable from other identifiable component clusters localized in the frontal, temporal, parietal, and occipital lobes active in other phases of the task. As such, while this is a preliminary analysis focusing on the mu rhythm only, studies in the future on larger sample sizes may reveal other physiological processes more closely related to the coordination of encoding, maintenance, and execution processes that also account for behavioral accuracy. Two limiting factors in the current study are the relatively small sample size and relatively high accuracy on the behavioral task, suggesting caution in interpreting correlations with behavioral accuracy. For those reasons, future studies of the mu rhythm with larger sample sizes should investigate tasks in which behavioral accuracy is decreased and has been reported to show differences in behavioral accuracy between groups (e.g., 7 syllable nonword repetition Bowers et al., 2018).

One prediction derived from neurobiological accounts of language is that a region at the parieto-temporal junction coordinates encoding, maintenance, and execution of nonword syllable sequences and is heavily involved in the acquisition of language (e.g., new speech sounds and words) in the preschool years (Hickok and Poeppel, 2007; Hickok et al., 2011; Majerus, 2013; Choo et al., 2016). Previous studies using the ICA approach have reported evidence of a posterior temporal lobe alpha rhythm with spatial, sensory, and sensorimotor functions consistent with the proposed function of the parieto-temporal junction in perception and production (Jenson et al., 2015; Bowers et al., 2019). Further, maintenance of syllable sequences may also be modulated by attentional control in prefrontal regions (D’Esposito, 2007; Majerus, 2013; Bowers et al., 2018). Interestingly, it has been proposed that stuttering may be characterized by deficits in sensorimotor timing that are modulated by ongoing cognitive-emotional states in prefrontal-basal ganglia networks (Chang et al., 2019). Thus, future studies using larger sample size and connectivity analyses between component clusters (e.g., phase coherence) have the potential to identify other time-sensitive processes proposed to be critical both for language acquisition and the sensorimotor control of speech (Bowers et al., 2019). In the future, signal separation approaches like the one employed in the current analysis (Delorme et al., 2012) or other approaches (e.g., Cheveigne et al., 2019) may be applied to prospective studies of recovery and persistence in preschool-age CWS and to differences in interactions between general cognitive capacities and sensorimotor control in AWS (Bowers et al., 2018).

## Summary

The ICA/time-frequency approach implemented to date that identifies and temporally decomposes EEG mu rhythms has yielded interesting findings that we believe contribute to the understanding of the neurophysiology of stuttering. Their sources and the sensitivity of its constituent frequency bands for capturing and differentiating between a broad array of sensorimotor and basal ganglia functions bestow mu rhythms with strong suitability for research in stuttering. Though much is already known about neural speech-related aspects of stuttering, the additional temporal and spectral resolution offer novel windows into the specific underpinnings of disfluency. Furthermore, they also provide a valuable means of linking the cognitive differences associated with stuttering to the underlying deficits that impact speech. While we continue to pursue and espouse this line of research, it is important to point out some current limitations and future directions.

## Limitations

Not all participants contributed usable mu components to the group analyses. The reduced subject contribution is common in EEG research (Nyström, 2008; Bowers et al., 2013), and is linked to the use of standard head models. Specifically, the stringent inclusion criteria employed in the current work require mu components to be localized to accepted generator sites, and the inability of standard head models to account for individual anatomic variability (von Ellenrieder et al., 2009) leads to some components with mu-like features (e.g., arch-like wave shape) localizing outside accepted generator sites. This reduced proportion of contributing subjects is further exacerbated by the age- and sex-matching of PWS and TFS to ensure the validity of statistical comparisons. If one member of a pair does not produce a usable mu component, data from both members are discarded. The use of individual head models in future studies is expected to address this limitation, increasing the proportion of contributing subjects and providing a fuller picture of the heterogeneity present across participants. A final potential limitation of the ICA methodology more broadly is differences across studies in preprocessing pipelines (e.g., filtering and IC selection). We suggest, following others, that increased use of automatized preprocessing methods will further facilitate replicability across studies and the increased use of EEG database repositories (Bigdely-Shamlo et al., 2015).

Another potential barrier to making use of the mu rhythm and EEG data more broadly is the inherent challenge of collecting high-quality EEG data from preschool-age children. Lengthy experimental protocols like those used in the EEG investigations described in this review may not be directly translated to experiment protocols for preschool-age participants and stimuli often require adaptation (Usler and Weber-Fox, 2015). Analysis of the mu rhythm in young children may require cross-sectional or longitudinal designs as the mu rhythm is known to change over the course of development and an adult-like mu rhythm may not be present in very young children (Thorpe et al., 2016). Special attention would need to be given to the contribution of movement artifact from young children even with the use of ICA to identify and separate artifacts from neural source data.

A further limitation of the presented data is the absence of a significant correlation to stuttering severity. Thus, while mu spectra and oscillatory activity clearly differentiate TFS and PWS, the precise manner in which the observed neural differences give rise to the speech disruptions characteristic of the disorder remains unclear. The few studies that have examined neural oscillations in relation to stuttered and fluent speech have suggested that differences in power and phase coherence precede stuttered speech (Sengupta et al., 2017) that may be variable across individuals (Myers et al., 2018). The presence of mu spectral and oscillatory differences in the absence of stuttered speech suggests that they represent a core neural impairment underlying the disorder, though stuttering is also shaped by life experience (Connery et al., 2019) and influenced by a number of other cortical and subcortical mechanisms (Alm, 2004). To date, a comprehensive neural framework describing how each of the distinct neural circuits implicated in stuttering operates together to give rise to the totality of the disorder remains elusive. Thus, while mu spectra and oscillatory activity holds promise for probing sensorimotor and basal ganglia influences on stuttering in real-time, findings must be interpreted within the larger context of all neural data regarding stuttering and merged into a comprehensive neural framework.

## Future Directions

To date, our measures have focused on comparisons of mu rhythm oscillations in AWS to TFS. Better separation of cause and effect associated with group differences will be achieved *via* recordings from CWS. In order to accomplish this, it is necessary to further refine data collection and analysis techniques. Data collection can be enhanced by using more child-friendly protocols. Use of beamforming (Cohen, 2015) or joint decorrelation analysis to target specific regions of interest or to identify a signal subspace of interest, in addition to ICA for denoising raw signals is likely to help identify mu rhythm activity more effectively in children. A comparison of component processing methods using the same experimental data may also help to cross-validate and compare findings from the ICA approach with other methods.

While mu rhythm measures continue to offer promise for investigation in stuttering, it also is necessary to extend measures of oscillatory activity to other regions of the brain such as regions of sensorimotor integration in posterior temporal lobes and inferior parietal lobes. Oscillations from the temporal lobe alpha rhythms have shown to be effective for capturing speech induced auditory suppression (Jenson et al., 2015) and there we have shown preliminary evidence of this activity differing in individuals who stutter (Saltuklaroglu et al., 2018b). With measures from multiple brain regions involved in stuttering, it will also be possible to capture real-time measures of functional connectivity (Delorme et al., 2011) showing how the transmission of neural information differs between stuttering and non-stuttering brains in a variety of speech and cognitive tasks.

## Data Availability Statement

The datasets generated for this study are available on request to the corresponding author.

## Ethics Statement

The studies involving human participants were reviewed and approved by the University of Tennessee, Idaho State University, and University of Arkansas Institutional Review Boards. The patients/participants provided their written informed consent to participate in this study.

## Author Contributions

DJ, AB, DH, and TS contributed to the content of this manuscript.

## Conflict of Interest

The authors declare that the research was conducted in the absence of any commercial or financial relationships that could be construed as a potential conflict of interest.

## Abbreviations

ANOVA: analysis of variance
ASR: artifact subspace reconstruction
AWS: adults who stutter
CV: consonant-vowel
CWS: child/children who stutter(s)
EEG: electroencephalography/electroencephalographic
EMG: electromyography/electromyographic
ERP: event-related potential
ERSP: event-related spectral perturbation
fMRI: functional magnetic resonance imaging
IC: independent component
ICA: independent component analysis
MARA: multiple artifact rejection algorithms
MEG: magnetoencephalography
PWM: phonological working memory
PWS: person(s) who stutter(s)
SLP: syllable load performance
TFS: typically fluent speaker
OASES: Overall Assessment of the Speaker’s Experience of Stuttering.
